# Investigation on the Attention Deficit Hyperactivity Disorder Effect on Infatuation and Impulsivity in Adolescents

**DOI:** 10.3389/fnbeh.2019.00137

**Published:** 2019-07-09

**Authors:** Lorrayne Stephane Soares, Danielle de Souza Costa, Leandro Fernandes Malloy-Diniz, Marco Aurélio Romano-Silva, Jonas Jardim de Paula, Débora Marques de Miranda

**Affiliations:** ^1^Programa de Pós-Graduação em Medicina Molecular, Universidade Federal de Minas Gerais, Belo Horizonte, Brazil; ^2^Department of Mental Health, Faculdade de Medicina, Universidade Federal de Minas Gerais, Belo Horizonte, Brazil; ^3^Department of Psychology, Faculdade de Ciências Médicas de Minas Gerais, Belo Horizonte, Brazil; ^4^Department of Pediatrics, Universidade Federal de Minas Gerais, Belo Horizonte, Brazil

**Keywords:** attention deficit/hyperactivity disorder, ADHD, impulsivity, passionate love, infatuation

## Abstract

**Introduction:**

In this study, we proposed to investigate the association between infatuation/passionate love and impulsivity in a context of potential high impulsivity: adolescents with attention deficit and hyperactivity/impulsivity (ADHD) diagnosis compared with typically developing adolescents.

**Methods:**

Impulsivity was understood as an exploratory and a sensation seeking behavior, a trend to engage in novel and exciting activities, and was evaluated using the UPPS Impulsive Behavior Scale. Eighty-one adolescents from 13-to-18 years old with and without ADHD diagnosis were compared regarding infatuation intensity, behavioral impulsivity, and social and educational profiles.

**Results:**

After correlation analysis, we found association between higher scores on the infatuation intensity with fewer years of formal education, heightened urgency and sensation seeking. On the other hand, using the generalized equation model, we showed that the association of passionate love with behavioral urgency and sensation seeking did not change in the presence of the ADHD diagnosis.

**Conclusion:**

The understanding of the relationship of impulsivity with infatuation might help to clarify why some population groups show an increased risk for many negative social outcomes.

## Introduction

Human pair-bonding and reproduction are complex cross-cultural phenomena involving physiological, cognitive, and emotional changes with high impact on behavior (Hatfield and Rapson, 1993; Fisher et al., 2016). Specifically, the passionate love strategy may have increased human offspring survivability as partners focusing time and energy on one another would probably rear a child as a team. Infatuation, also known as passionate love, is the falling in love or simply an intense amorous feeling for one individual. Passionate love is the first phase of a romantic relationship, a phase of positive emotions, but also a negative and stressful one if marked by break-ups and life dissatisfaction. Passionate love has been argued as a “natural addiction,” including aspects such as obsessive and intrusive thinking, euphoria, and craving (Fisher et al., 2016). Furthermore, the process of falling in love engages brain reward system areas involved in chemical or behavioral addiction, specifically dopamine pathways, with the ventral tegmental area (VTA) consistently associated with intense infatuation (Fisher et al., 2016).

Attention-deficit/hyperactivity disorder (ADHD) is the most prevalent neurodevelopmental disorder in childhood and adolescence, affecting as much as 5% of young people around the world (Barkley et al., 2006; Polanczyk et al., 2007). ADHD is highly complex regarding its etiology but has a strong genetic base with evidence of reward processing alteration and dopaminergic dysfunction (Dalley and Roiser, 2012; Beauchaine et al., 2017). Impulsivity is a core deficit and an important long-term symptom of ADHD. Children with high levels of hyperactive-impulsive symptoms have higher rates of academic drop out and fewer years of education (Fredriksen et al., 2014). Adolescents with ADHD have greater rates of car accidents and delinquency, poorer performance in educational and employment settings, lower occupational status, and social problems with family members and peers (Polanczyk et al., 2007; Bussing et al., 2010). Additionally, ADHD negatively impacts romantic relationships, which is evidenced by lower marital satisfaction, higher rates of divorce (Biederman et al., 1993; Murphy and Barkley, 1996), an aggressive attitude toward others (Wymbs et al., 2012), and poorer conflict resolution (Canu and Carlson, 2007). Antisocial and impulsivity-driven behaviors may partly explain many social and academic outcomes (Fredriksen et al., 2014).

Impulsivity can be considered action with no conscious thinking, a behavior delivered without enough self-control, and a tendency to respond without planning (Moeller et al., 2001). Different approaches have been used to evaluate several aspects of impulsivity, ranging from self-report questionnaires to cognitive measures (Salgado et al., 2009). One of such aspects related to impulsivity is associated with an exploratory behavior, a trend to engage in novel and exciting activities, and a sensation-seeking behavior. Sensation seeking may serve an adaptive purpose, increasing the chances of reproductive success and food obtention (Irwin and Millstein, 1986; Spear, 2000), but also can lead to negative outcomes (Whiteside and Lynam, 2001). Relative to human development, sensation seeking likely peaks in adolescence (Zuckerman, 1974; Roth et al., 2005). In adolescence, youngsters start to engage in new and exciting experiences and begin to expand and have more complex social interactions and relationships. Adolescence is a vulnerable phase for risk-taking. Statistics point to a peak in dangerous activities, such as car accidents, auto and hetero-aggression, drug and alcohol abuse, unprotected sex, and a rise in psychopathologies’ rate (Steinberg and Monahan, 2007; Casey et al., 2008). The high sensitivity to incentives and contexts related to impulsivity in adolescence was associated with an imbalance in the self-control circuitry modulated by dopamine (Casey, 2015).

Based on that, this study was conceived to better understand the relationship between impulsivity and the intensity of romantic love in conditions of high impulsivity (ADHD and adolescence). Specifically, this study aimed to investigate a putative association between ADHD, infatuation intensity, and behavioral impulsivity, since they appear to share some neural substrates. Then, we sought to evaluate a possible moderation effect of ADHD on the relationship between infatuation and impulsivity in adolescence.

## Methods

### Participants and Study Design

This was a transversal study of adolescents (*n* = 81) aged 13-to-18 years old, being 55 (67,9%) girls and 26 (32,1%) boys, with 51 (63%) of them presenting a typical development and 30 (37%) with ADHDs. Of these, 18 (60%) were only inattentive, 5 (16.7%) were only hyperactive/impulsive and 7 (23.3%) had a combined profile. Typically-developing participants were from two local urban schools or from a collaborative school from a nearby city, and adolescents with ADHD were recruited in an impulsivity and attention research center. The ADHD screening was performed with the MTA-SNAP-IV (Mattos et al., 2006), which shows good accuracy in detecting this condition among Brazilian children and adolescents (Costa et al., 2018). Adolescents with typical development were classified based on parents reporting a lack of history of psychiatric or neurological disease and may not have borderline or clinical scores in the internalizing and externalizing scales of the Child Behavior Checklist (parent form) (Achenbach et al., 2011). The study was approved by the local ethics board. All the volunteers gave written informed consent and their main caregiver consented for participation. The study is in accordance with the Declaration of Helsinki.

### Instruments

#### Juvenile Love Scale (JLS)

To measure infatuation/passionate love intensity, we used the Brazilian version of the Juvenile Love Scale (JLS) (Hatfield and Young, 1998). The JLS is composed of 30 items rated on a nine points-Likert scale with higher scores indicating a higher passionate love intensity. The JLS access cognitive, emotional, and behavioral features of passionate love such as intrusive thinking and idealization of the other, attraction toward the partner (especially sexual), positive and negative feelings, physiological arousal, physical proximity, and be available to the other (Hatfield and Sprecher, 1986; Cacioppo et al., 2012). We used the Brazilian version short version of JLS, containing 15 items, which was previously validated to our culture (Soares et al., 2017). In this sample, JLS internal consistency was higher than 0.90 suggesting good reliability.

Juvenile Love Scale scores, obtained by summing all items, provides a global measure on how much infatuated the respondent is. In the original study, Hatfield and Young (1998) described five different categories based on percentile data, ranging from “the thrill is gone,” the lower level of passionate love, and “wildly, even recklessly, in love,” the higher one. However, since our sample is relatively small, we divided our participants in only two groups (more infatuated and less infatuated groups), based on the sample median JLS score.

#### UPPS Impulsive Behavior Scale (UPPS)

We administered the UPPS Impulsive Behavior Scale as a behavioral measure of impulsivity (Whiteside and Lynam, 2001). The scale was adapted and validated to the Brazilian context (Sediyama et al., 2017). The UPPS is a self-report scale with 45 items addressing four behavioral dimensions of impulsivity: (1) urgency, a tendency to act precipitously under distress or extreme negative emotions; (2) (lack of) premeditation or acting without thinking; (3) (lack of) persistence, related to the ability of remain focused on a task; and (4) sensation seeking, a tendency to engage in novel and exciting experiences (Whiteside and Lynam, 2001; Cyders et al., 2007). The scale is presented in a Likert-type format ranging from 1 to 4: (1) strongly agree, (2) partially agree, (3) partially disagree, and (4) strongly disagree. Higher scores are suggestive of higher impulsivity. UPPS internal consistency in this sample was higher than 0.90.

#### Sociodemographic Characteristics

Information about adolescents’ age, sex, and education was given by parents. Participants’ socioeconomic status (SES) was characterized by the *Brazilian Economic Classification Criteria (CCEB)* which provides evidence about purchasing power and general situation of the households through questions about possession of durable goods and educational level of the head of the household. Scores can vary from 0 to 100 and fall in one of six socioeconomic strata: A (monthly household income estimation of U$ 6464.42), B1 (monthly household income estimation of U$ 2863.93), B2 (monthly household income estimation of U$ 1501.60), C1 (monthly household income estimation of U$ 837.14), C2 (monthly household income estimation of U$ 502.90), and DE (monthly household income estimation of U$ 237.68) (ABEP, 2008). Higher scores suggest better SES. In this sample, participants had the following economic classifications: 4 (9%) A, 7 (16%) B1, 15 (35%) B2, 9 (21%) C1, 7 (16%) C2, 1 (2%) DE (ABEP, 2008).

### Statistical Procedures

All analyses were performed with SPSS 22.0. We conducted descriptive statistics and correlational analysis to investigate variables distribution and their associations. Then, we tested whether significant associations between passionate love and impulsivity measures would change depending on group diagnosis. General Linear Models were built independently for each significant impulsivity measure associated with passionate love (dependent variables). Main effects and group-by-passionate love interaction were computed controlling for significant demographic differences between typically developing and ADHD adolescents.

## Results

Table 1 shows descriptive information regarding adolescents’ age, education, and socioeconomic situation. Additionally, descriptive information was given for passionate love intensity through the JLS and UPPS factors (i.e., urgency, lack of premeditation, lack of perseverance, and sensation seeking). Typically developing adolescents were mostly girls (*n* = 43) and ADHD participants mostly boys (*n* = 17). We found no group differences regarding sex in any measure of ADHD, impulsivity or in passionate love (all *p* > 0.05).

**TABLE 1:** Participants’ characteristics and group comparisons.

	**Control**		**ADHD**		**Comparison**		
	***M***	***SD***	***M***	***SD***	***U***	***Z***	**Parameter**
Age	16.0	1.3	15.3	1.4	546.5^*^	−2.2	–
Socioeconomic status	1	15.4	39.6	19.3	603.5	−1.4	–
Education	10.0	1.0	10.0	1.2	405.5^∗∗^	−3.3	–
Inattention (SNAP-IV)	10.5	10.4	26.0	9.8	1430.0^∗∗^	−4.9	>15^1^
Hyperactivity/impulsivity (SNAP-IV)	4.1	3.7	12.2	6.1	1513.0^∗∗^	−3.7	>10^1^
ODD symptoms (SNAP-IV)	4.0	4.4	10.1	6.1	1609.0^∗∗^	−2.4	>7^1^
Urgency (UPPS)	28.6	6.8	30.9	8.0	654.5	−1.1	29.0 (6.0)^2^
Premeditation (UPPS)	24.5	8.0	25.5	7.2	688.0	−0.7	24.0 (5.0)^2^
Perseverance score (UPPS)	23.1	6.3	24.5	6.8	688.0	−0.7	21.0 (5.0)^2^
Sensation seeking (UPPS)	31.7	7.2	34.9	6.8	583.0	−1.8	34.0 (7.0)^2^
Juvenile Love Scale (15 items)	76.3	31.6	77.7	35.2	738.5	−0.3	.

SE, standard error; U, Mann–Whitney *U*-test; Z, difference between standard scores. ^*^p = 0.05; ^∗∗^*p* < 0.01. ^1^Cutoff for ADHD according to Costa et al. (2018), ^2^We found no Brazilian normative values for adolescents in UPPS, so we added parameters – Mean (SD) based in d’Acremont and Van der Linden (2005) community study. This second parameter should be interpreted carefully, since the referred study was conducted with French Adolescents, a different culture from our sample.

We found an association between higher scores on the JLS, heightened urgency and sensation seeking (Table 2). We didn’t find any association between participants’ sex, education, and socioeconomic level and love intensity. The general linear model (see Table 3) showed that the association of passionate love with behavioral urgency and sensation seeking did not change depending on ADHD diagnosis. Additionally, we tested if any of these associations were moderated by the age of the participant, stratified by the samples median (16 years). We found no interaction between age, diagnosis, passionate love on any impulsivity measure.

**TABLE 2:** Participants’ correlations among Passionate Love and Impulsivity measures on adolescence.

**Variable(s)**	**1**	**2**	**3**	**4**	**5**	**6**	**7**	**8**
(1) Passionate Love (JLS)	–							
(2) Age (years)	–0.03	–						
(3) Education	–0.21	0.71^∗∗^	–					
(4) Socioeconomic level (CCEB)	0.02	0.03	0.06	–				
(5) Urgency (UPPS)	0.28^*^	0.13	–0.10	–0.20	–			
(6) Premeditation (lack of) (UPPS)	–0.01	–0.01	–0.02	–0.07	0.37^∗∗^	–		
(7) Perseverance (lack of) (UPPS)	–0.17	0.08	–0.01	–0.10	0.37^∗∗^	0.60^∗∗^	–	
(8) Sensation seeking (UPPS)	0.26^*^	–0.17	–0.19	–0.08	0.29^∗∗^	0.09	–0.11	–

JLS, Juvenile Love Scale; CCEB, Brazilian Economic Classification Criteria (higher scores suggest higher socioeconomic situation); UPPS, UPPS Impulsive Behavior Scale. ^*^p < 0.05; ^∗∗^*p* < 0.01.

**TABLE 3:** Effect of Passionate Love on adolescents’ Impulsivity measures depending on ADHD status.

**Outcome**	**Predictor**	***F***	***df***	***p*-value**	**$\beta_{p}^{2}$**
Urgency (UPPS)	Passionate Love (JLS)	4.14^*^	1	0.045	0.051
	Group	0.34	1	0.561	0.004
	Group^*^Passionate Love (JLS)	1.63	1	0.130	0.021
Sensation seeking (UPPS)	Passionate Love (JLS)	4.41^*^	1	0.039	0.054
	Group	0.76	1	0.386	0.010
	Group^*^Passionate Love (JLS)	0.16	1	0.205	0.021

JLS, Juvenile Love; UPPS, UPPS Impulsive Behavior Scale; *df*, degrees of freedom. ^*^p < 0.05.

Table 4 shows the participants’ scores on all four impulsivity domains, divided into infatuated (above average and below average on JLS scores), and then categorized in ADHD group (6 or more hyperactivity and/or inattentive symptoms on SNAP-IV) and control group (5 or less hyperactivity and/or inattentive symptoms on SNAP-IV).

**TABLE 4:** Description of participants’ scores on the UPPS scale stratified by JLS scores.

	**Less infatuated group**		**More infatuated group**	
	**Control**	**ADHD**	**Control**	**ADHD**
**Variable(s)**	**Mean (SD)**	**Mean (SD)**	**Mean (SD)**	**Mean (SD)**
Urgency	30,0 (8,7)	33,1 ( ± 7,3)	28,7 ( ± 6,1)	27,5 ( ± 6,8)
Premeditation	24,8 ( ± 9,2)	25,1 ( ± 8,3)	24,4 ( ± 7,1)	25,4 ( ± 6,5)
Perseverance	20,5 ( ± 6,1)	24,4 ( ± 7,2)	25,0 ( ± 6,1)	24,2 ( ± 6,2)
Sensation seeking	35,4 ( ± 6,4)	34,9 ( ± 6,1)	30,0 ( ± 7,3)	33,4 ( ± 7,3)

*SD, standard deviation.*

## Discussion

To the best of our knowledge, this is the first study investigating the possible effects of romantic love on impulsivity in adolescents and its relationship with ADHD symptoms. Our results showed that love affects sensation seeking and urgency, suggesting that passionate love intensity may exert some influence on these aspects of impulsivity. There is also a correlation between love and urgency, as well as there is between love and ADHD symptoms. These results point toward a positive direction relationship, which means that the more passionate, more sensation seeking behaviors are exhibited, and more reckless they tend to act when under negative emotions.

Self-control appears to be an important factor for maintaining a long-term relationship. According to early studies, cognitive control would predict behaviors which could contribute to a lasting relationship, including staying faithful, resist flirting and being forgiving (Finkel and Campbell, 2001; Ritter et al., 2010). In the early stages of love, however, the impulsivity related to sensation-seeking could be beneficial (Van Steenbergen et al., 2014). Sensation seeking refers to individual high motivation for novelty and intense and unusual sensory experiences (Norbury and Husain, 2015). Our results of higher rates of sensation seeking behaviors among the more infatuated subjects could indicate an openness for new habits and experiences, which would allow incorporating the other in one’s life (Aron and Aron, 1986). Unbalanced top-down cognitive control might enhance impulsive activity (Hofmann et al., 2009), just as observed in addiction (Dalley et al., 2011). Some authors have suggested that passionate love is a “natural addiction” since they share these brain processes drive, such as impulsivity (Fisher et al., 2016). Indeed, the striatal dopamine release on the in-love brain is similar to the process involved in addiction, with an enhancement of reward regulation networks, and is also related to sensation seeking (Fisher et al., 2005; Frascella et al., 2010; Xu et al., 2011). This dopaminergic modulation could change the balance between striatal and prefrontal connections, favoring an increase of impulsivity in the early stages of passionate love (Cools, 2008; Van Steenbergen et al., 2014). Additionally, the hypothalamic pituitary adrenal (HPA) axis increases its activity during the early stages of passionate love, indicating a high-stress level (Marazziti and Canale, 2004). Stress can be seen as an altered mood state and insecurity (Stárka, 2007; Berscheid, 2010), too typical of the early stages of passionate love, contributing to non-rational behavior in a love situation. According to our results, more infatuated individuals may tend to act rashly (urgency), especially in contexts associated with the loved one.

We found no moderating effect of ADHD diagnosis over love and impulsivity interaction, indicating that passionate love intensity seems to affect ADHD and typically-developing adolescents in the same way. Despite the changes in cortico-striatal pathways associated with diverse forms of impulsivity in ADHD, there was no additive role of the disorder in adolescents’ passionate love influence on urgency or sensation seeking tendencies.

Our study has limitations that must be addressed. One of the study’s limitations was the sample size, which was insufficient to detect small effect sizes. In this sense, more discrete associations may not be perceived in our data. The control group was not interviewed by a child psychiatrist due to the context of their data collection (schools). To minimize this bias, we used the CBCL scale as a screening tool, an instrument that shows high concordance with a clinical interview when responded by the participants’ parents (Brasil and Bordin, 2010). We also did not have detailed data on participants’ relationship status, even though they could just think about someone when informing scores in the JLS. We had no information about how long possible couples were together, partners’ age and SES, neither on participants’ pubertal development. Of the ADHD sample, 60% were only inattentive, 16.7% were only hyperactive/impulsive, and 23.3% had a combined presentation profile. The inattentive predominance might partially explain why there was no difference in UPPS impulsive scores between adolescents with and without ADHD in our sample. As it is inherent to any correlation approach, we cannot determine whether passionate love increases urgency and sensation seeking behaviors when one is in love, or if adolescents for whom extreme emotions act like a boost to impulsiveness, or that are inclined to live exciting new experiences, get to experience more intense infatuation.

Intense passionate/romantic love is a near-universal human phenomenon. The turmoil that accompanies adolescence may serve as a motivation enhancer to courtship attraction and the pursuing of a mating partner, which can lead to a love that is returned or rejected. Anyhow, passionate love likely increases activity related to impulsivity for better or for worse. The understanding of the relationship of impulsivity with infatuation might help to clarify why some population groups show an increased risk for many negative social outcomes.

## Data Availability

The datasets generated for this study are available on request to the corresponding author.

## Ethics Statement

This study was carried out in accordance with the recommendations of the local ethical board (UFMG/Plataforma Brasil) with written informed consent from all subjects. All subjects and primary caregivers gave written informed consent in accordance with the Declaration of Helsinki. The protocol was approved by the UFMG Ethics committee.

## Author Contributions

LS conceptualized and designed the study, contributed to data collection, conducted the initial analyses, drafted the initial manuscript, and reviewed and revised the manuscript. DC and JdP conceptualized and designed the study, conducted additional analyses, contributed to interpretation of data analysis, drafted the initial manuscript, and reviewed and revised the manuscript. LM-D, MR-S, and DdM obtained funding, conceptualized and designed the study, contributed to interpretation of data analysis, and reviewed and revised the manuscript. DdM also drafted the manuscript. All authors approved the final manuscript as submitted. All authors agreed to be accountable for all aspects of the work, including the accuracy and integrity of this study.

## Conflict of Interest Statement

The authors declare that the research was conducted in the absence of any commercial or financial relationships that could be construed as a potential conflict of interest.
